# Thermal Analysis of Heat Transfer from Catheters and Implantable Devices to the Blood Flow

**DOI:** 10.3390/mi12030230

**Published:** 2021-02-25

**Authors:** Hossein Zangooei, Seyed Ali Mirbozorgi, Seyedabdollah Mirbozorgi

**Affiliations:** 1 Mechanical Engineering Department, University of Birjand, Birjand 9717434765, Iran; zangooei93@gmail.com (H.Z.); samirbozorgi@birjand.ac.ir (S.A.M.); 2 Electrical and Computer Engineering Department, University of Alabama at Birmingham, Alabama, AL 35294, USA

**Keywords:** implantable devices, catheters, ultrasound imaging, blood flow, heat transfer, thermal analysis, numerical analysis, non-Newtonian fluid

## Abstract

Implantable devices, ultrasound imaging catheters, and ablation catheters (such as renal denervation catheters) are biomedical instruments that generate heat in the body. The generated heat can be harmful if the body temperature exceeds the limit of almost 315 K. This paper presents a heat-transfer model and analysis, to evaluate the temperature rise in human blood due to the power loss of medical catheters and implantable devices. The dynamic of the heat transfer is modeled for the blood vessel, at different blood flow velocities. The physics and governing equations of the heat transfer from the implanted energy source to the blood and temperature rise are expressed by developing a Non-Newtonian Carreau–Yasuda fluid model. We used a Finite Element method to solve the governing equations of the established model, considering the boundary conditions and average blood flow velocities of 0–1.4 m/s for the flow of the blood passing over the implanted power source. The results revealed a maximum allowable heat flux of 7500 and 15,000 W/m^2^ for the blood flow velocities of 0 and 1.4 m/s, respectively. The rise of temperature around the implant or tip of the catheter is slower and disappeared gradually with the blood flow, which allows a higher level of heat flux to be generated. The results of this analysis are concluded in the equation/correlation $T=310+\frac{H}{3000}\left(1+e^{-\sqrt{7V}}\right)$, to estimate and predict the temperature changes as a function of heat flux, *H*, and the blood flow velocity, *V*, at the implant/catheter location.

## 1. Introduction

The human body is a thermodynamic system that regulates the internal body temperature by physiological actions [1]. Any thermal disorder of the human body will be regulated to a constant body core temperature of 309.65 K (36.5 °C) naturally (T(K) = T(°C) + 273.15). At rest, the core body temperature has a narrow range between 309.15 K (36 °C) and 311.15 K (38 °C), and it temporarily increases up to 314.15 K (41 °C) with heavy exercise [2,3,4]. To regulate core temperature, the body’s metabolic heating and cooling mechanisms make a balance with external heat increment or loss, with the help of evaporation, radiation, convection, and conduction processes. The liver, brain, heart, and muscles are the primary sources of body heat generation. Although the body can be adapted to a great range of external temperature changes, including hot, humid, or arid conditions, a high temperature can cause serious hyperthermia, resulting in body stress, the danger of injury, and death. To avoid damage, the body core temperature must not exceed the normal narrow range, while localized heating can be increased outside of this narrow range temporarily [2,3,4,5,6,7,8,9].

**Figure 1:**
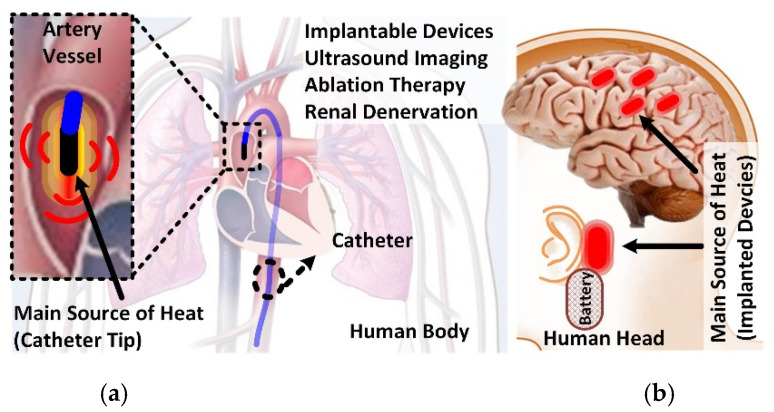
The conceptual schematic of (**a**) ultrasound imaging device, implanted Intra-Cardiac Echocardiography (ICE)/Intra-Vascular Ultrasound (IVUS) catheter, located in the blood vessel, and (**b**) brain implantable devices.

The use of catheters and implantable devices is becoming popular for diagnostics and therapy purposes, and overheating in the surrounding tissue caused by these devices is a concern for such technologies [10,11,12,13,14,15,16,17,18]. The conceptual schematics of such applications are shown in Figure 1.

The consumed power will eventually be converted to heat inside the body and dissipates in the body tissue and blood (regulated by the metabolic cooling mechanism of the body). These devices are imaging and therapeutic catheters, implantable microchips, telemetry coils, etc., which can be located everywhere inside the body. Imaging Intra-Cardiac Echocardiography (ICE) and Intra-Vascular Ultrasound (IVUS) catheters [19,20,21,22,23,24], ablation catheters (employing electricity to heat or burn tissue locally as a treatment) such as Renal Denervation (RDN) catheters used for resistant hypertension [25], and millimeter-sized implantable devices for Deep Brain Stimulation (DBS) and neural recording microchips [10,11,12,13,14,15,16,17,18] are examples of widely used bio-implantable devices. These devices have electronic circuits and need electrical power for operation, requiring thermal safety considerations during the design process. Table 1 presents the specifications (regarding the heat generation issues) for these biomedical applications, indicating the power consumptions, sizes of the devices, locations in the body, and allowable temperature rise.

Ultrasound imaging is a well-known approach that employs an array of transducers to propagate ultrasound waves and uses the reflected waves to produce interior body images. The ICE and IVUS catheters are advanced types of ultrasound devices, in which high-voltage drivers and low-noise amplifier electronics are located at the tip of the catheters to transmit ultrasound waves and record the reflection for imaging [19,20,21,22,23,24]. The power consumption of such catheters is high compared with other implantable applications (Table 1), and a cooling mechanism must be considered in many cases.

Ablation catheters are developed and used to destroy abnormal tissue (ablation therapy) [26,27]. Cardiac ablation catheters are used for the treatment of cardiac arrhythmias (heart rhythm problems) by permanently destroying discrete foci or eliminating tissue that is pivotal for sustaining reentrant circuits in the myocardium [26]. Similarly, the RDN catheters are designed for conducting procedures to treat resistant hypertension [28]. Biological and clinical trials have recently proved RDN procedures’ efficacy for lowering blood pressure as a minimally invasive approach [29]. By disabling specific renal artery nerves (renal sympathetic denervation), the blood pressure will be decreased [30]. The RDN catheters deliver thermal energy to denervate the renal artery’s adventitia after being inserted through the femoral artery. For fast ablation without critical cellular degeneration, the temperature of the artery wall near the RDN catheter’s electrode should be within the range of 338.15–348.15 K (65–75 °C), while the cellular vaporization occurs at 373.15 K (100 °C) [26,27]. It is necessary to understand the heat transfer mechanism (from the catheter/implant to the surrounding blood and tissue layers) and estimate the temperature rise accurately for designing reliable catheters and reducing the risks of overheating. To avoid overheating risks and causing excessive damage, temperature sensors and closed-loop control mechanisms are considered in the catheter design [25,27].

The use of implantable devices, including neuroprosthetic devices for restoring and rehabilitating disabled sensory and motor functions, DBS devices, millimeter-sized implants, cochlear implants, retinal implants, limb movement systems, bladder control implants, chronic pain control devices, etc., is increasing, and the thermal concerns of such heat producing devices need to be addressed [10,11,12,13,14,15,16,17,18,31,32]. For example, the temperature rise in the eyes, due to the use of retinal stimulator implants (in the sensitive surrounding environment), must be evaluated to address safety concerns for long-time operations of the implant [16,33,34]. Although implantable devices have small power consumptions, the thermal effect is a vital issue to be studied for continuous operations, such as DBS devices [35] and neural recording implantable systems.

In this study, using numerical analysis, a probe/implant is modeled and the temperature increment rate in blood flow is simulated and studied to provide a design rule for addressing the safety concerns. The remainder of this paper is organized as follows. Section 2 presents the details of the modeling of a probe (implant or catheter tip) and governing equations to analyze the temperature rise. Section 3 covers the software modeling and simulation results. Section 4 includes our discussion on a design rule for the future development of such implants, followed by a conclusion in Section 5.

## 2. Method: Problem Description and Modeling

### 2.1. Geometry and Governing Equations

**Figure 2:**
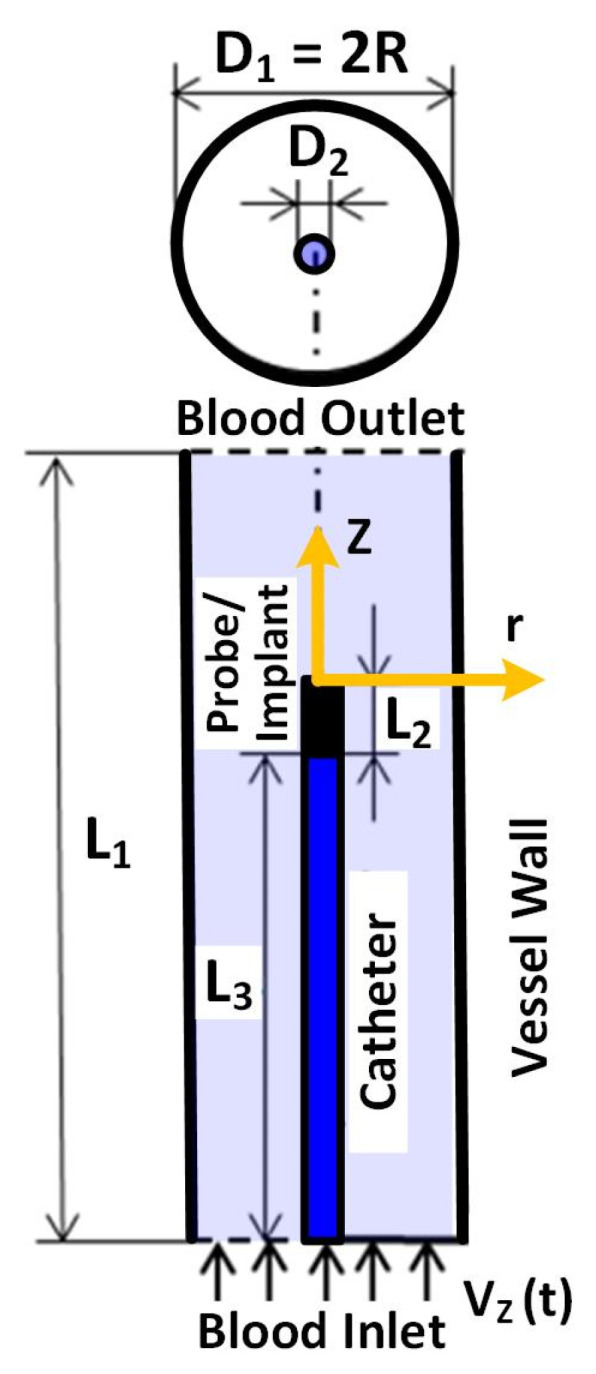
Schematic of BVI system, including the blood vessel, catheter, and probe/implant.

We have modeled a 3D geometry of a Blood-Vessel-Implant (BVI) system. The schematic of the BVI system is shown in Figure 2, indicating (1) the inlet and outlet blood flows, (2) the catheter and its tip (probe/implant), and (3) the vessel. This figure includes the details of the BVI system’s dimensions, in which the *D*_1_ and *L*_1_ are the inner diameter and the length of the modeled cylindrical blood vessel (human vein), respectively. The *D*_2_ is the outer diameter of the catheter (length: *L*_3_) and implant/probe (length: *L*_2_). We have considered a rigid property for modeling the vessel wall of the BVI system. In the modeled system, the blood flow is entered from the bottom (inlet) toward the Z direction. The primary geometry of the BVI system and the corresponding dimensions are reported in Table 2.

The *D*_1_ is varied for the developed model, to cover different vessel sizes in the human body, from 5 to 20 mm [36].

We utilized a set of Navier–Stokes (NS) equations, [16,37], including continuity, momentum, and energy equations for modeling and simulating the blood flow and heat transfer in the BVI system. This set of equations expresses the differential forms of the three conservation principles (the conservation of mass, momentum, and energy) in fluid flow systems, which are as follows:

(1) $$\frac{\partial \rho }{\partial t}+\nabla \cdot (\rho \;\vec{V})=0$$

(2) $$\rho [\frac{\partial \vec{V}}{\partial t}+(\;\vec{V}\cdot \nabla )\;\vec{V}]=-\nabla p+\nabla \cdot \;\tau_{i\;j}+f$$

(3) $$\rho c_{p}(\frac{\partial T}{\partial t}+\vec{V}\cdot \nabla T)=-\nabla \cdot \;q+Q$$

where $\vec{V}=V_{x}i+V_{y}j+V_{z}k$ is the fluid flow velocity vector, *ρ* is the fluid density, *p* is the pressure, *τ_ij_* is the shear stress tensor (for an incompressible flow: *τ_ij_ = µ*$\dot{\gamma }$, where *µ* is the dynamic viscosity and $\dot{\gamma }$ is the shear rate), *f* is the volumetric body forces, ***c_p_*** is the specific heat capacity at constant pressure, *T* is the temperature, *Q* is the heat source, *k* is the thermal conductivity of the fluid, and *q* is the heat flux (which *q = −k*∇T, expressing the Fourier’s Law of Heat Conduction).

A non-Newtonian fluid does not follow Newton’s law of viscosity (constant viscosity independent of stress). While in non-Newtonian fluids, viscosity can change under force to be either more liquid or more solid [38]. Blood’s behavior against shear stress, *τ_ij_*, is considered as a Non-Newtonian fluid. Therefore, we used a Non-Newtonian fluid model to develop the NS equations for the BVI system’s blood model. To model the blood fluid using COMSOL Multiphysics^®^ software (COMSOL Inc., Burlington, MA, USA), we used a Carreau–Yasuda Non-Newtonian fluid model and applied it to the NS equations. In a Newtonian incompressible fluid flow, the stress and strain rates have a linear relationship with a constant *μ*. In contrast, this relationship is nonlinear for Non-Newtonian fluids, where *μ* expressed by Equation (4).

(4) $$\frac{\mu -\mu_{\infty }}{\mu_{0}-\mu_{\infty }}={\left[1+{\left(\lambda \dot{\gamma }\right)}^{\alpha }\right]}^{(n-1)/\alpha }$$

In this model, the $\mu_{\infty }$,$\;\mu_{0}$, and λ are the viscosity at an infinite shear rate, the zero-shear-rate viscosity, and the relaxation time (seconds), respectively. In Equation (4), the parameters *α* and *n* control the blood dilatation behavior due to shear stress [38].

### 2.2. Boundary Conditions and Numerical Solution Method

**Figure 3:**
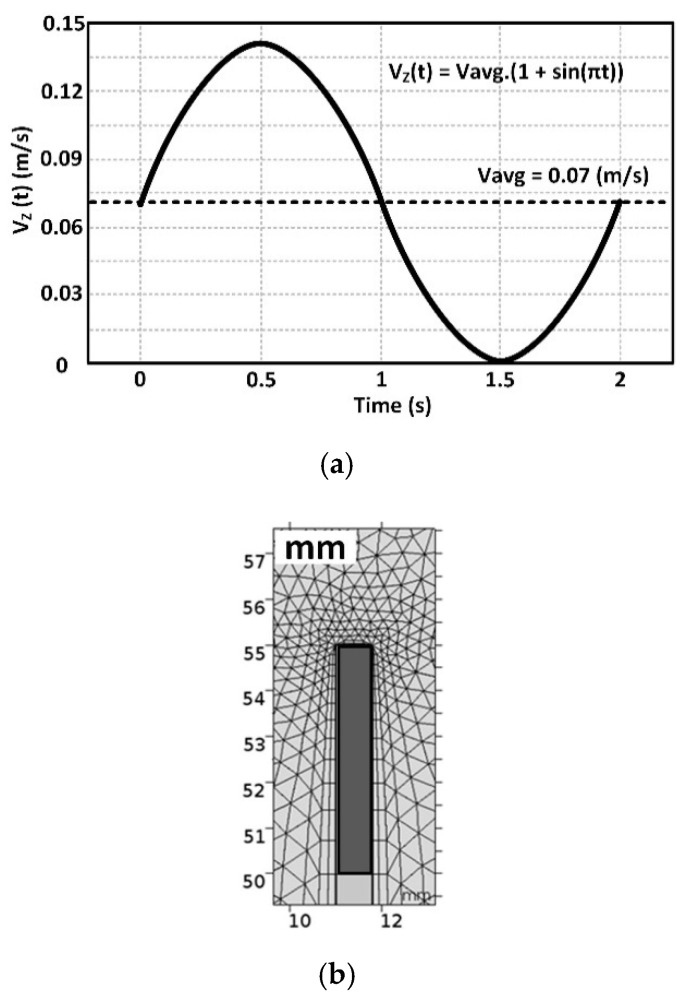
(**a**) The applied blood flow velocity profile as a function of time at the inlet of the blood vessel, and (**b**) the schematic of the BVI system indicating the solution mesh (grid).

Considering the periodic nature of the blood flow in the human body, we have generated a sinusoidal blood flow and applied it to the input of the system (shown in Figure 2). One period of the applied blood flow velocity profile to the BVI system is shown in Figure 3a as a function of time.

This periodic function of the blood flow velocity is summarized in Equation (5):

*V_Z_(t)* = *V_avg_* [1 + *sin(πt)*] (5)

where *V_avg_* (at the inlet of the vessel) is the average blood flow velocity [39], and the time period of the blood flow velocity fluctuation is considered 2 s. The boundary condition for the blood flow velocity at the internal walls of the vessel and the surface of the heat source (the probe/implant or tip of the catheter) is a non-slip condition. The boundary conditions in the outlet of the BVI system can be expressed with the derivative form of the velocity in the Z-direction, which its derivation equals zero, $\frac{\partial V_{z}}{\partial z}=0$, [40]. Regarding the pressure boundary conditions, the pressure has been considered zero at the outlet due to (1) the incompressibility of the flow field, and (2) the principle of the pressure’s relativity. On the other hand, at the inlet and all the rigid boundaries, we have utilized the derivative condition of the pressure in all directions, which equals zero, $\frac{\partial p}{\partial n}=0$. Since blood fluid flow is incompressible, the effect of the pressure on the distribution of the temperature can be disregarded [40]. The incompressible flow implies that the density remains constant within a parcel of fluid that moves with the flow velocity. Additionally, in the modeled boundary conditions for the BVI system, we have considered an insulated condition (for simplicity) between (1) the vessel’s walls and the blood, and (2) the blood and the vessel’s walls. For the heat source walls, we used a constant heat flux condition. Regarding the temperature conditions of the model, the inlet is set to have a constant value (310.15 K), while for the outlet of the model, a derivative form of the temperature is considered. In addition, the physical effect of insulation is that no heat flows across the boundary (the temperature gradient is zero). In general, when fluid is flowing inside a tube under forced convection (fluid motion generated by an external source) under laminar flow conditions, constant heat flux and constant wall temperature conditions are demonstrated.

The flow geometry of the BVI system is the cylindrical space between the heat source and the vessel wall. A 3D grid, generated by the COMSOL software, is designated for modeling the BVI system. Figure 3b shows the 2D view of the BVI model and the developed primary mesh for simulation. The cells of the grid are square near the heat source region and triangular in distant points from the heat source region. COMSOL Multiphysics^®^ software provides a cross-platform for Finite Element analysis, solver, and multiphysics simulations [41]. Additionally, the COMSOL software is a commercial computational fluid dynamics (CFD) modeling simulation package. In this work, we have used this software to model and simulate the BVI system to study the dynamic and thermal performance and characteristics of the system. To model the BVI system, we limited the number of cells (of the mesh) to be no more than 300,000 units and the sizes of the cells with the range of 0.02 to 0.8 mm. We utilized the Generalized Minimal Residue (GMRES) algorithm of the COMSOL’s solver in this work.

### 2.3. Model Development

To develop the BVI system model, based on Equations (1)–(3), and estimate the heat transfer rate to the blood, we used the initial values of *ρ, c_p_*, and *k*, presented in Table 3 [42,43].

The BVI system’s primary geometry and its schematic are shown in Table 2 and Figure 2, respectively. The parameters of the Carreau–Yasuda model are presented in Table 4 for Equation (4) and then Equation (2) in the BVI model [44,45]. We substituted Equation (4) in Equation (2) (by calculating *μ* from Equation (4) and transferring it to Equation (2) using *τ_ij_ = μ*$\dot{\gamma }$, in an iterative process) and used the parameters of Table 4 to find the required momentum equation of non-Newtonian fluid for the Carreau–Yasuda model.

To complete the model and run the simulation, we determined an optimum time step required for our model among the three different time steps of $t_{d}=\frac{\Delta z^{2}}{\vartheta }$ (for the diffusion mechanism), $t_{c}=\frac{\Delta z}{V_{z}}$ (for the convection mechanism), and $t_{p}=\frac{1}{p/\rho }\frac{\Delta z}{V_{z}}$ (for the pressure mechanism), where ∆*z* is the average mesh size, *V_z_* is the flow average reference velocity (m/s), and $\vartheta =\frac{\mu }{\rho }\;$ kinematic viscosity. Since the $t_{c}=\frac{1}{Re}t_{d}$ and Re>1 (Re: Reynolds number), the $\;t_{c}<t_{d}$. The Reynolds number is $Re=\frac{\rho V_{z}D_{1}}{\mu }=\frac{V_{z}D_{1}}{\nu }$, where the *ρ* is the density of the fluid (SI units: kg/m^3^), $D_{1}$ is a characteristic dimension (m), *μ* is the dynamic viscosity of the fluid (Pa·s or N·s/m^2^ or kg/m·s), and *ν* is the kinematic viscosity of the fluid (m^2^/s) [43].

### 2.4. Validation of the Carreau–Yasuda Non-Newtonian Fluid Model for Blood

**Figure 4:**
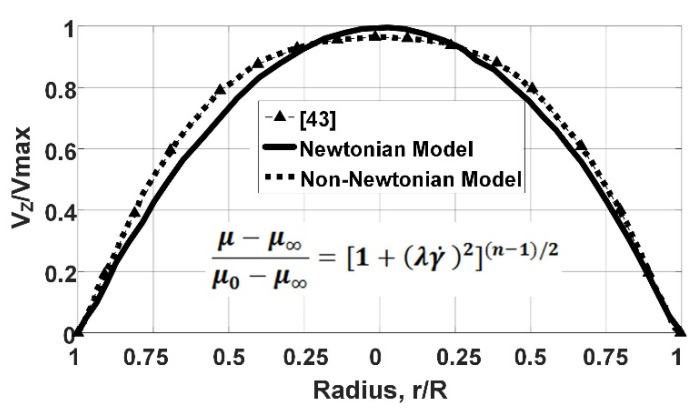
Comparison of Newtonian and Non-Newtonian fluids simulation results with experimental results from Reference [43].

To validate the blood flow model as a Carreau–Yasuda Non-Newtonian fluid flow, we analyzed the dynamical behavior of the fluid numerically, considering the Non-Newtonian, steady-state, and fully developed flow assumptions (without velocity fluctuations at the inlet). For this validation, a rigid tube, 20 mm (inner diameter) and 80 mm (length), is modeled as the vessel by COMSOL. Figure 4 presents the simulation results of the rigid tube with the Non-Newtonian fluid flow.

On the other hand, $t_{p}=\frac{1}{c_{s}^{2}}t_{c}$ (c_s_: sound speed) for Re>1 (~500, in the BVI system), which implies $\;t_{p}\ll t_{c}$. The $t_{p}\;$ provides the smallest time step, but the duration for running the simulation is dramatically increased by using $t_{p}$. The optimum time step calculated for the BVI model and simulation is the $t_{c}$ = 0.0066 s [36,46,47].

This figure shows the dimensionless velocity distribution of the flow as a function of the transverse cross-section of the tube/vessel, including (1) experimental results [43], (2) simulated Newtonian fluid model, and (3) simulated Non-Newtonian fluid model. The developed Non-Newtonian fluid model of this work is in great agreement with the experimental results presented by Shaik [43]. These experimental results [43] show the velocity profile within a vessel, and it is compared with the BVI model (simulated Newtonian and Non-Newtonian fluid models). This comparison confirms and validates that the developed non-Newtonian fluid model of blood flow in a circular rigid tube/vessel matches experimental values of the blood viscosity. It is worth mentioning that the Newtonian and Non-Newtonian fluid models have almost identical velocity values near the tube walls and maximum deviation in the center of the tube.

**Figure 5:**
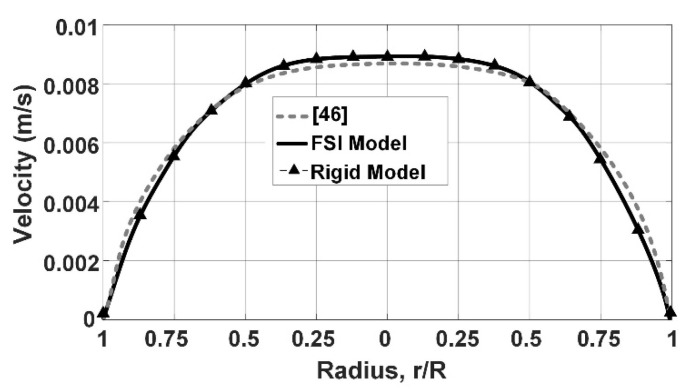
Comparison of Fluid–Structure Interaction FSI and rigid models with the experimental results from Reference [46]. The velocity of the blood flow, as a function of the transverse cross-section of the vessel.

On the other hand, we modeled and simulated both Fluid–Structure Interaction (FSI) and rigid tubes and compared the blood flow velocity results in the tubes with experimental results [43]. Figure 5 shows the velocity of the blood as a function of the transverse cross-section of the tubes. Our simulation results show an identical value of the gradient of the velocities for the FSI and rigid models at the vessel walls. Therefore, using rigid walls for modeling the vessel is acceptable for the developed BVI system.

## 3. Results: COMSOL Simulation

**Figure 6:**
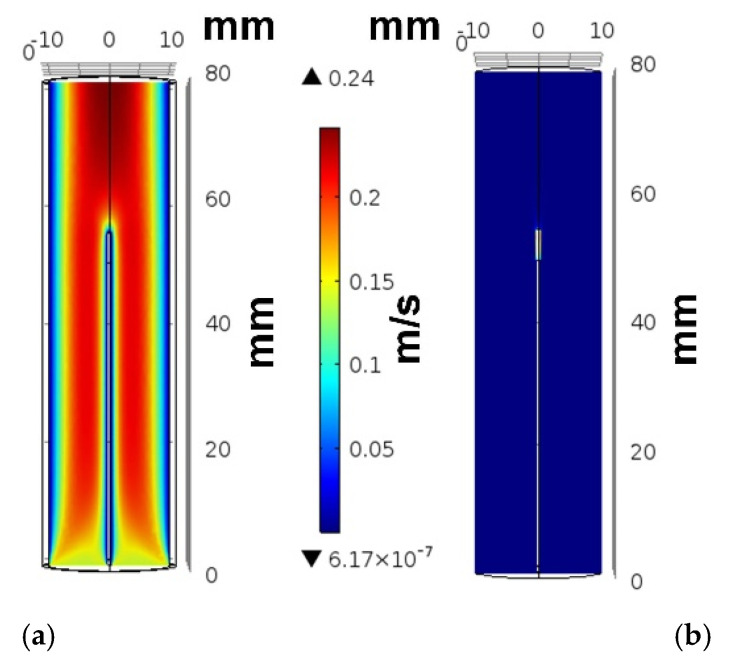
The simulated results of blood flow velocity contours in the BVI model for (**a**) maximum velocity at *t* = 0.5 s and (**b**) zero velocity at t = 1.5 s.

The BVI system of Figure 2 is modeled and simulated, using COMSOL. The velocity, pressure, and thermal simulation results of this system are presented and discussed for different vessel radiuses. The BVI model of Figure 2 is fed by the inlet blood flow velocity (min 0 m/s to max 0.14 m/s; average, 0.07 m/s) of Figure 3a, considering the vessel primary radius of R = 10 mm. Figure 6a,b shows the 2D cross-sections of the blood flow velocity, *V_z_* contours, for the 3D BVI model at *t* = 0.5 s (maximum velocity) and *t* = 1.5 s (zero velocity), respectively. This simulation confirms (1) the expected zero velocities at the walls, and (2) the blood flow velocity profile in the vessel is following the generated inlet sine wave velocity. As shown in Figure 6a, the velocity at the outlet has reached its maximum of 0.24 m/s, larger than the maximum inlet velocity, due to the considered zero pressure at the outlet of the vessel and generated pressure at the inlet by the applied velocity.

**Figure 7:**
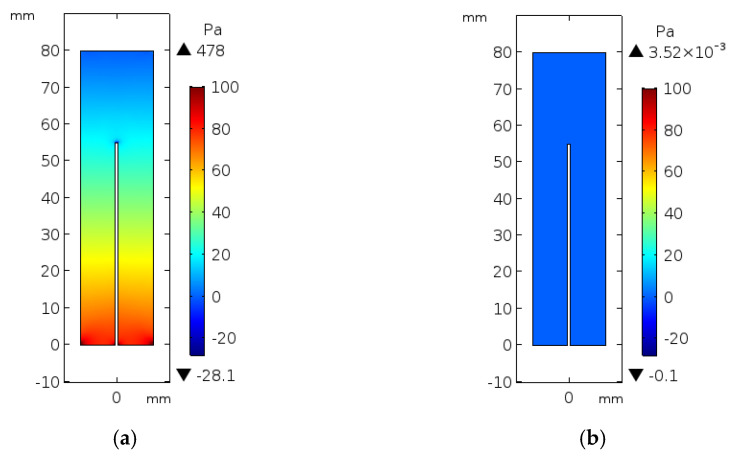
The simulated results of blood pressure contours in the BVI model for (**a**) maximum velocity at *t* = 0.5 s and (**b**) zero velocity at *t* = 1.5 s.

Figure 7 shows the 2D cross-sections of blood pressure contours in the vessel for the 3D BVI model. The pressure is generated by applying the velocity at the inlet of the modeled vessel while the outlet pressure is set zero to guarantee the production of the blood flow at the modeled short length of the vessel. Figure 7a,b shows the generated pressure contours at the maximum velocity (*t* = 0.5 s) and the zero velocity (*t* = 1.5 s), respectively. As shown in Figure 7a, the maximum pressure of 478 Pa is generated at the inlet of the vessel. Figure 6a and Figure 7a confirm the inverse relationship of the velocity and pressure at the inlet and outlet of the vessel.

**Figure 8:**
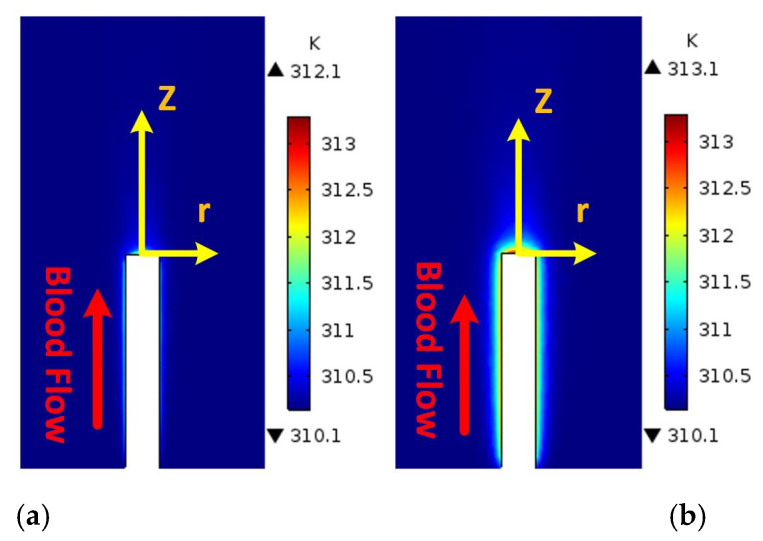
The simulated temperature for (**a**) maximum velocity at *t* = 0.5 s and (**b**) zero velocity at *t* = 1.5 s.

Figure 8 shows the 2D cross-sections of blood temperature contours in the vessel for the 3D BVI model. The temperature distribution is generated by the heat flux produced by the probe, set for the simulation to be 12,000 W/m^2^ (typical level of the heat flux generated by ultrasound catheters). In this case, the power consumption of the implant (the tip of the catheter or the probe/implant) equals 154 mW when its surface area equals 13.06 mm^2^. Figure 8a,b shows the generated temperature at the maximum velocity (*t* = 0.5 s) and the zero velocity (*t* = 1.5 s), respectively. As shown in Figure 8a, the minimum temperature of 312.1 K is obtained at the maximum velocity (0.14 m/s at the inlet). Figure 8b shows the maximum temperature of 313.1 K at the minimum blood flow velocity (0 m/s at the inlet). Figure 6a and Figure 8a confirm the inverse relationship of the blood flow velocity and the temperature.

**Figure 9:**
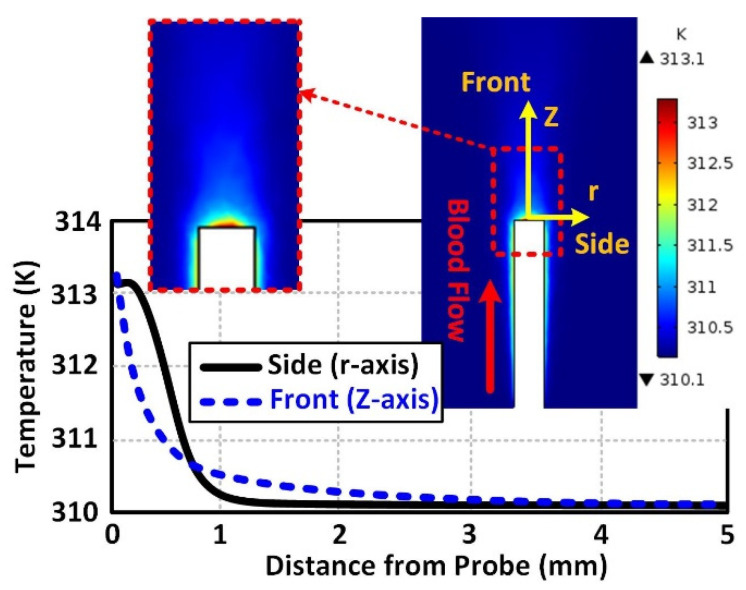
Temperature propagation as a function of distance from the surface of the probe for two main directions (front and side).

Figure 9 illustrates the close-up of the simulated temperature 2D profile (Figure 8b) and the temperature distribution pattern. The temperature variation as a function of distance from the probe is extracted and plotted for the front (Z-axis) and side (r-axis) of the probe/implant. In this plot, the distance zero indicates the surface of the probe. In this simulation, the Z-axis is the blood flow velocity direction. The maximum temperature rise is accrued at the surface of the probe when the blood flow velocity drops to its minimum level.

**Figure 10:**
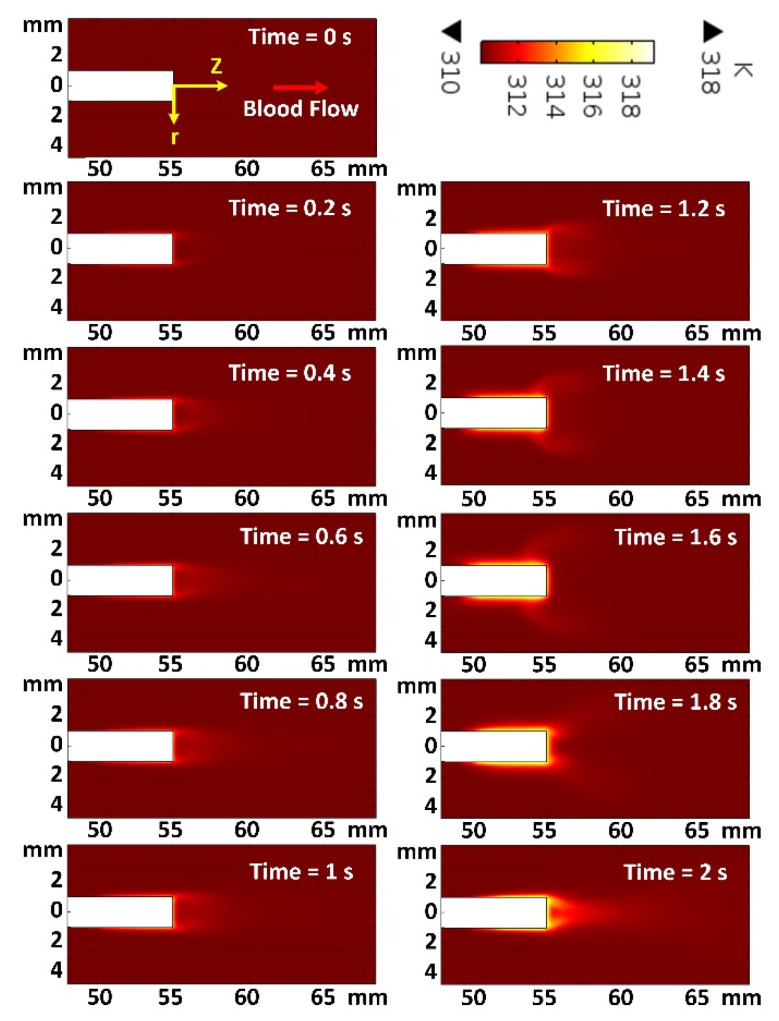
Temperature contours in the 2D plane for maximum heat flux case, at Z = 55 mm and different moments. Distribution of temperature in the radial direction for the cross-section of Z = 55 mm at different moments.

Figure 10 provides the temperature distribution details at different times from *t* = 0 s to *t* = 2 s (covering a complete cycle of a heartbeat period), with a 0.2 s step size. In a steady-state condition, it is expected to have a maximum temperature rise at minimum blood flow velocity and vice versa (which is not the case for this work). The simulation results presented in Figure 10 indicates that the maximum temperature (in front of the probe) is achieved at *t* = 2 s, while the velocity is zero at *t* = 1.5 s. The inward current continues after blood flow ceases, resulting in a greater accumulation of heat and a rise in temperature. It shows a 90° lag between the maximum temperature rise and the minimum of the blood flow velocity, which can be modeled based on the current and voltage relationship of a capacitor in an electrical circuit.

**Figure 11:**
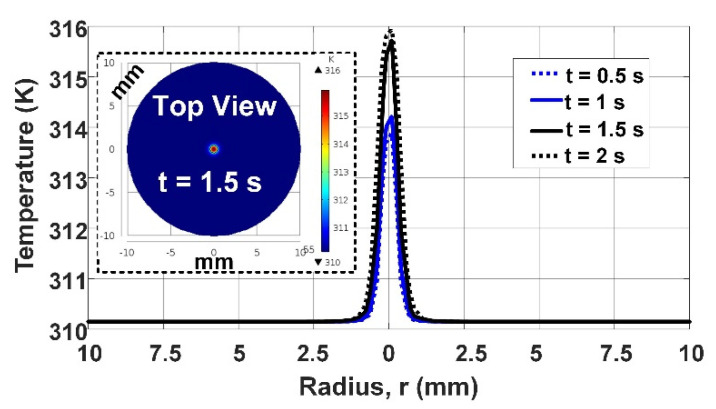
The simulated results of blood temperature at 12,000 W/m^2^ of heat flux rate as a function of the radius of the vessel for different blood velocities at the indicated times. The inset includes the temperature contours at a cross-section of the vessel (at the tip of the probe) for *t* = 1.5 s.

**Figure 12:**
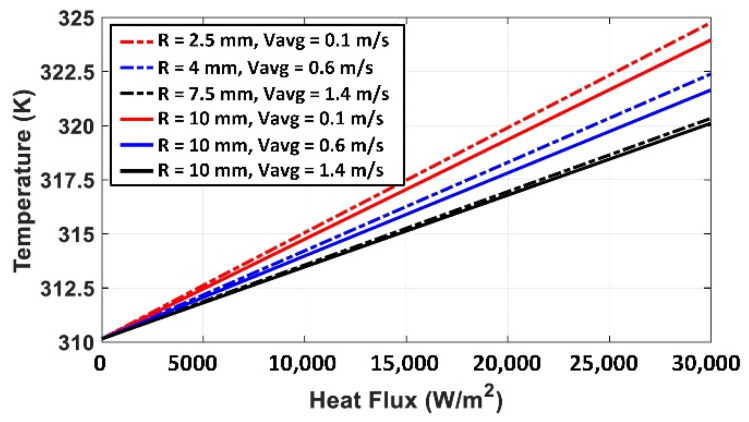
The simulated results of blood temperature as a function of the heat flux for average blood flow velocity of 0.1 to 1.4 m/s and different vessel’s radiuses; *R* = 2.5 mm–*R* = 10 mm.

Figure 11 shows the simulated results of blood temperature as a function of the diameter of the vessel, while the heat flux is set at 12,000 W/m^2^ rates. It includes the temperatures for different times of *t* = 0.5 s, *t* = 1 s, *t* = 1.5 s, and *t* = 2 s, indicating the blood velocities of 0.14, 0.07, 0, and 0.07 m/s, respectively. The inset figure of Figure 11 illustrates the temperature contours of the blood vessel at the tip of the probe, used to capture (1) the temperature profile vs. radius of the vessel for Figure 11, and (2) the maximum temperature values for the curves in Figure 12.

To characterize the BVI system, we changed the heat flux of the probe/implant from 0 to 30,000 W/m^2^ and monitored the temperature. This simulation is repeated for different levels of blood velocities and different radiuses of the blood vessels. The results are shown in Figure 12; the temperature (K) is a function of heat flux, indicating a linear relationship between the heat flux and the temperature rise in the blood. Based on the results of Figure 12, this linear relationship between the temperature and heat flux is always valid at different blood velocities and different radiuses of blood vessels. These results indicate a temperature rise of less than 5 K when the average blood flow velocity changed from 1.4 to 0.1 m/s (at the heat flux rate of 30,000 W/m^2^). On the other hand, a temperature rise of 1.1 K is obtained by reducing the inner radius of the blood vessel from 10 to 2.5 mm at 30,000 W/m^2^ (red curves in Figure 12). It is worth mentioning that the temperature rise has a direct and linear relationship with heat flux (temperature increases by increasing the heat flux), while the temperature is reduced by increasing the blood flow velocity or increasing the radius of the blood vessel, non-linearly.

We changed the blood vessel’s radius in the proposed BVI model and captured the temperature rise. The results are presented in Table 5. The radiuses of the 2.5, 4, 7.5, and 10 mm are selected based on human body physiology; the names of the blood vessels are indicated in the table (the blood vessel length: 8 cm). Different blood flow velocities are considered for these simulations based on the blood vessels’ locations in the body and the average blood flow velocities of an adult male. Table 5 has reported the maximum temperature rise in the blood vessels due to the heat flux of 12,000 W/m^2^. These results show that the temperature rise is higher in a blood vessel with a smaller radius at an identical blood flow velocity.

## 4. Discussion and Limitations

**Figure 13:**
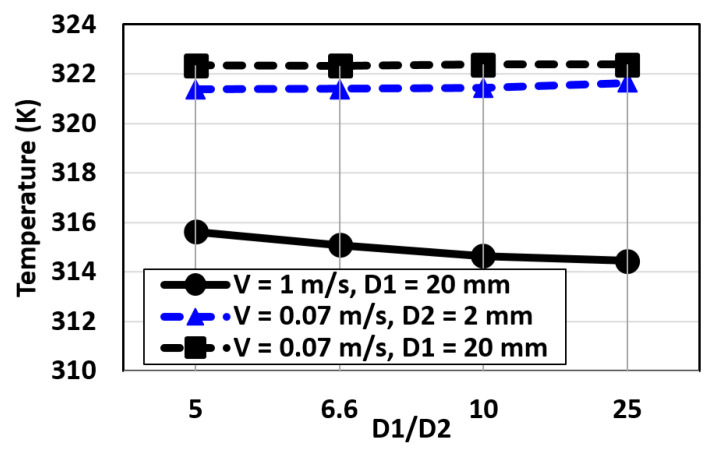
The temperature changes at the tip of the probe as a function of D1/D2 for blood velocity of 0.07 and 1 m/s: D1 of 10, 13.3, 20 mm; and D2 of 0.8, 2, 3, and 4 mm (H = 12,000 W/m^2^).

The proposed BVI model is simulated for different values of the blood flow velocities and heat fluxes using COMSOL. Figure 13 shows the temperature changes at the tip of the probe as a function of D1/D2. For this figure, the diameters of the blood vessel (D1) and probe (D2) are changed, and the obtained temperatures are reported based on the ratio of D1/D2. This simulation includes the results for V = 0.07 m/s and V = 1 m/s. Based on these results, the temperature changes as a function of D1/D2 can be ignored for low blood velocities and be less than 1 K at high blood velocities.

**Figure 14:**
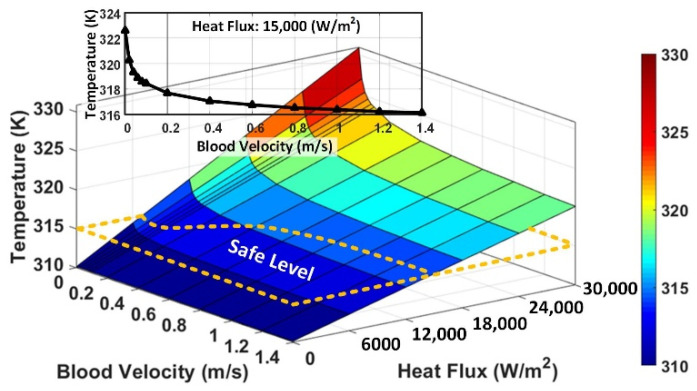
The simulated results of blood temperature as a function of the heat flux and blood flow velocity, indicating the safe temperature level (blood vessel radius: 10 mm). Inset: the temperature as a function of velocity at 15,000 W/m^2^.

To generalize this study, we changed the blood flow velocities from 0 m/s (i.e., brain implant devices) to 1.4 m/s (i.e., probes in the vessels near the heart), and heat fluxes from 0 to 30,000 W/m^2^ (~3 times larger than the maximum heat flux generated by the imaging catheter). We extended this range up to 100,000 W/m^2^, to cover the ablation catheter applications. The results are provided in Figure 14, indicating the temperature rise as a function of the blood flow velocity and heat flux. The natural body (blood) temperature range is 309.65–310.65 K, and the tolerable temperature rise is almost 315 K [48,49]. When the blood flow velocity is higher, heat flux (power consumption of the implant) can be higher while the temperature rise is still under the safe level, as is shown by the dashed lines in Figure 14.

Therefore, the maximum allowable heat flux of 7500 and 15,000 W/m^2^ are obtained for the blood flow velocity of 0 and 1.4 m/s, respectively. With a higher blood flow velocity, the temperature rise around an implant is slower and disappears gradually, allowing implanting a design with a higher power consumption level inside the body. The dominant heat transfer mechanism in this system is convection (circulation or movement of the heated parts of a liquid). At the lower blood flow velocities, the rate of the temperature rise increases exponentially due to the reduction of the convection. Figure 14’s inset illustrates the temperature as a function of velocity, at 15,000 W/m^2^, highlighting the exponential increment of the temperature when the velocity is closing to zero, suggesting ($T\propto 1/e^{f\left(V\right)}$), while the temperature rise is proportional with the heat flux linearly (T∝H). Based on the results presented in Figure 14, we derived/correlated an equation, Equation (6), to estimate and predict the level of the temperature, *T* (K), changes as a function of the heat flux, *H* (W/m^2^), and the blood flow velocity, *V* (m/s), at the location of the implant/probe.

(6) $$T=310+\frac{H}{3000}\left(1+e^{-\sqrt{7V}}\right)$$

Equation (6) helps extend the range of the velocity and heat flux to estimate the temperature rise for the applications with higher power ranges, such as tumor ablation. Equation (6) is plotted in 3D, and the temperature is shown as a function of the heat flux and blood flow velocity, up to reach a temperature rise near 380 K (burning and evaporating body cells and tissues).

**Figure 15:**
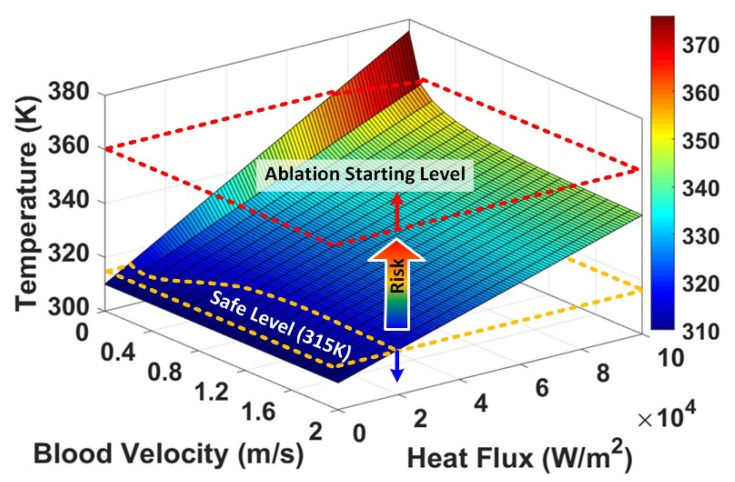
The blood temperature as a function of the heat flux and blood flow velocity, indicating the safe temperature level and ablation temperature level (vessel radius: 10 mm).

Figure 15 shows the results with a heat flux range of 0–100,000 W/m^2^ and the blood flow velocity range of 0–2 m/s. As indicated, the temperature rise under 315 K is a safe level, and the risk of damaging the tissue layers increases at the higher temperatures. If the purpose of using the implant/probe is to burn the tissue layers (such as killing cancerous tumor cells), the temperature must be raised to the levels above 360 K [50].

The required heat flux for ablation purposes (*H_Ablation_*) can be calculated from Equation (6); assuming a blood flow velocity of zero, *V* = 0, based on the zero exponent rule, e^0^ = 1, we can substitute $e^{-\sqrt{7V}}{|}_{V=0}=1$ in Equation (6), we will have the following:

(7) $$H_{Ablation}=\frac{Power\;\left(W\right)}{Surface\;\left(m^{2}\right)}=1500\left(T_{Ablation}-310\right)$$

The *T_Ablation_* can be defined based on a designated treatment plan (considering the dimension of the target area, duration of ablation, etc.), usually above 360 K. For example, if the required heat flux for ablation equals 135,000 W/m^2^ (with an effective heating surface of 2 cm^2^ or 0.002 m^2^) to reach 400 K (based on Equation (7)), the required power equals the product of the heat flux and the surface of the probe/implant, 27 W.

**Figure 16:**
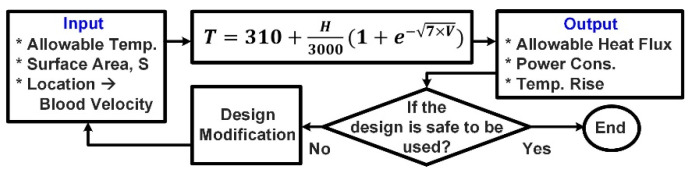
The proposed design safety-check flowchart for inserted devices with electrical power consumptions.

Figure 16 shows a design safety check (design rule) flowchart for calculating temperature rise and addressing the safety concerns of any designs (implants and probes/catheters) with electrical power consumption inside the body (human or animals). This flowchart helps the designers and researchers to estimate the temperature rise (or allowable power consumption) for implantable devices, study the safety issues and concerns accurately, and apply the required modifications to their design, without the need for safety simulations.

The BVI model is based on intravascular procedures for imaging, and it can be applied to intracardiac ablations and mm-sized implants applications for studying heat transfer, too. The provided results and formulas support different applications with different levels of generated heat fluxes (up to 100,000 W/m^2^) and blood flow velocities (0–2 m/s). In the BVI model, the body of the catheter does not contribute to delivering heat in the system, and the sizes of the implants are limited to the mm-sized implants with a surface area of <100 mm^2^ and low blood flow velocities (near 0 m/s).

## 5. Conclusions

We developed and presented a heat transfer model for electronic devices designed to be operated inside the human body. These electronic devices produce heat by consuming electric power. Using the proposed BVI model, we analyzed and evaluated the temperature rise and changes in human blood due to the power loss of medical catheters. The heat generated in the body by such an inserted electronic energy source can be harmful if the body temperature exceeds the limit of 315 K for a long time. To better understand the dynamics of the heat transfer function in the blood vessel, the system was modeled by developing a Non-Newtonian Carreau–Yasuda fluid model. Therefore, the temperature rise was expressed by using the Finite Element method, considering the boundary conditions and the flow of the blood passing over the inserted power source, covering a blood flow velocity range of 0–1.4 m/s. Based on the presented study, we found maximum allowable heat fluxes of 7500 W/m^2^ for 0 m/s of blood flow velocity and 15,000 W/m^2^ for 1.4 m/s. The presented analysis has suggested $T=310+\frac{H}{3000}\left(1+e^{-\sqrt{7V}}\right)$ for estimating the temperature changes as a function of heat flux and the blood flow velocity at the location of the probe/catheter. As expected, a higher level of heat flux can be generated by the probe/catheter if we have blood flow around the device, since the temperature rise is slower and disappears gradually.

## Figures and Tables

**Table 1:** The summary of specifications for the implantable devices and catheters that generate heat.

Devices	Applications	Surface Area (mm^2^)	Power Consumption	Produced Heat Flux (W/m^2^)	Allowable Temperature (K)	Location/Blood Flow Level	References
ICE Catheters	Imaging	<130	<1000 mW	<8000	~315 (41.85 °C)	Blood Vessel/High	[19,20,21,22,23]
IVUS Catheters	Imaging	<65	<700 mW	<12,000	~315 (41.85 °C)	Blood Vessel/High	[19,20,21,22,23]
Cardiac Arrhythmias, RDN Catheters	Ablations	<315	2.5–30 W	<100,000	~338.15–353.15 (65-80 °C)	Heart/High Kidney/Low	[25,26]
Brain Implants	Recording and Stimulation	mm-sized: <100	<5 mW	<500	~310.65 (37.5 °C)	Brain/Low (<0.02 cm/s)	[14,31,32,35]

RDN, Renal Denervation.

**Table 2:** The dimension of the modeled Blood-Vessel-Implant (BVI) system of Figure 2.

*L*_1_ (mm)	*L*_2_ (mm)	*L*_3_ (mm)	*D*_1_ (mm)	*D*_2_ (mm)
80	5	50	5 < D_1_ < 20	0.8

**Table 3:** The properties of human blood [43].

Property	Abbreviation	Unit	Blood	Vessel
Density	ρ	kg/m^3^	1060	960
SHC CP ^1^	*C_P_*	J/kg·K	3900	-
Thermal Conductivity ^2^	*k*	W/m·K	0.5	-
Young Modulus ^3^	*E*	N/m^2^	-	10^7^
Poisson Ratio ^4^	*Nu*	1	-	0.3

^1^ Specific Heat Capacity at Constant Pressure. ^2^ Thermal conductivity is expressed as watts per meter-kelvin. ^3^ Young’s Modulus measures the tensile stiffness of solid material and is the slope of the linear part of the stress–strain curve expressed as Newton per square meter. ^4^ Poisson Ratio is a measure of expansion or contraction of material in directions perpendicular to the direction of loading.

**Table 4:** The parameters of the Carreau–Yasuda model.

$\mu_{\infty }(\mathrm{Pa}\cdot s)$	$\mu_{0}(\mathrm{Pa}\cdot s)$	*n*	*α*	λ (s)
0.0345	0.56	0.3568	2	3.313

**Table 5:** Simulation results: the maximum temperature rise for different blood vessel radiuses at the constant heat flux of 12,000 W/m^2^ (vessel length: 8 cm).

Vessel Type [36]	Radius	Average Velocity	Temperature (K)	Schematic
Femoral Artery Carotid Artery	2.5 mm	0.1 m/s	313.12	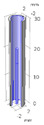
Femoral Artery Carotid Artery	4 mm	0.6 m/s	312.59	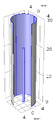
Abdominal AortaInferior Vena Cava	7.5 mm	1.4 m/s	312.22	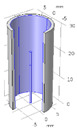
Ascending Aorta Descending Aorta Abdominal Aorta	10 mm	0.1 m/s 0.6 m/s 1.4 m/s	312.92 312.45 312.15	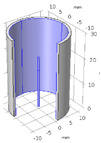

## References

[1] Djongyang N., Tchinda R., Njomo D. (2010). Thermal comfort: A review paper. Renew. Sustain. Energy Rev..

[2] Katić K., Li R., Zeiler W. (2016). Thermophysiological models and their applications: A review. Build. Environ..

[3] Choi J.H., Loftness V. (2012). Investigation of human body skin temperatures as a bio-signal to indicate overall thermal sensa-tions. Build. Environ..

[4] Witzmann F.A., Rhoades R.A., Bell D.R. (2009). Temperature regulation and exercise physiology. Medical Physiology: Principles for Clinical Medicine.

[5] Hochstein S., Rauschenberger P., Weigand B., Siebert T., Schmitt S., Schlicht W., Převorovská S., Maršík F. (2016). Assessment of physical activity of the human body considering the thermodynamic system. Comput. Methods Biomech. Biomed. Eng..

[6] Boregowda S.C., Choate R.E., Handy R. (2012). Entropy Generation Analysis of Human Thermal Stress Responses. ISRN Thermodyn..

[7] Yi L., Fengzhi L., Yingxi L., Zhongxuan L. (2004). An integrated model for simulating interactive thermal processes in hu-man–clothing system. J. Thermal Biol..

[8] Holopainen R. (2012). A Human Thermal Model for Improved Thermal Comfort.

[9] Fiala D., Lomas K.J., Stohrer M. (1999). A computer model of human thermoregulation for a wide range of environmental conditions: The passive system. J. Appl. Physiol..

[10] Ruggera P.S., Witters D.M., Von Maltzahn G., Bassen H.I. (2003). In vitro assessment of tissue heating near metallic medical implants by exposure to pulsed radio frequency diathermy. Phys. Med. Biol..

[11] Nutt J.G., Anderson V.C., Peacock J.H., Hammerstad J.P., Burchiel K.J. (2001). DBS and diathermy interaction induces severe CNS damage. Neurology.

[12] Wolf P.D., Reichert W.M. (2008). Thermal Considerations for the Design of an Implanted Cortical Brain–Machine Interface (BMI). Indwelling Neural Implants: Strategies for Contending with the In Vivo Environment.

[13] Lazzi G. (2005). Thermal effects of bioimplants. IEEE Eng. Med. Boil. Mag..

[14] Kim S., Tathireddy P., Normann R.A., Solzbacher F. (2007). Thermal Impact of an Active 3-D Microelectrode Array Implanted in the Brain. IEEE Trans. Neural Syst. Rehabil. Eng..

[15] Pennes H.H. (1948). Analysis of Tissue and Arterial Blood Temperatures in the Resting Human Forearm. J. Appl. Physiol..

[16] DeMarco S.C., Lazzi G., Liu W., Weiland J.D., Humayun M.S. (2003). Computed SAR and thermal elevation in a 0.25-mm 2-D model of the human eye and head in response to an implanted retinal stimulator-Part I: Models and methods. IEEE Trans. Antennas Propag..

[17] Chai R., Zhang Y. (2017). Recursive Multi-step Prediction of Bioimplants Thermal Effect. Struct. Health Monit..

[18] Singh V., Roy A., Castro R., McClure K., Dai R., Agrawal R., Greenberg R.J., Weiland J.D., Humayun M.S., Lazzi G. (2008). On the Thermal Elevation of a 60-Electrode Epiretinal Prosthesis for the Blind. IEEE Trans. Biomed. Circuits Syst..

[19] Gurun G., Tekes C., Zahorian J., Xu T., Satir S., Karaman M., Hasler J., Degertekin F.L. (2014). Single-chip CMUT-on-CMOS front-end system for real-time volumetric IVUS and ICE imaging. IEEE Trans. Ultrason. Ferroelectr. Freq. Control..

[20] Tekeş C., Karaman M., Degertekin F.L. (2010). Simulated annealing based optimization of dual-ring arrays for for-ward-looking IVUS and ICE imaging. IEEE Int. Ultrason. Symp..

[21] Jung G., Tekes C., Rashid M.W., Carpenter T.M., Cowell D., Freear S., Degertekin F.L., Ghovanloo M., Degertekin L. (2018). A Reduced-Wire ICE Catheter ASIC With Tx Beamforming and Rx Time-Division Multiplexing. IEEE Trans. Biomed. Circuits Syst..

[22] Herickhoff C.D., Wolf P.D., Smith S.W., Grant G.A., Britz G.W., Wilson C.M. (2010). Intracranial dual-mode IVUS transducer for image-guided brain therapy preliminary experiments. IEEE Int. Ultrason. Symp..

[23] Tekes C., Xu T., Carpenter T.M., Bette S., Schnakenberg U., Cowell D., Freear S., Kocaturk O., Lederman R.J., Degertekin F.L. Real-time imaging system using a 12-MHz forward-looking catheter with single chip CMUT-on-CMOS array. Proceedings of the 2015 IEEE International Ultrasonics Symposium (IUS).

[24] Mirbozorgi S.A., Tekes C., Pirouz A., Kocaturk O., Lederman R., Ghovanloo M., Degertekin F.L. A feasibility study for MRI guided CMUT-based intracardiac echocardiography catheters. Proceedings of the 2017 IEEE International Ultrasonics Symposium (IUS).

[25] Yang J., Kim S., Hwang G., Kwon K., Jeon S., Bae H.-M. (2019). Reference-Less Time-Division Duplex Transceiver IC for a Renal Denervation System. IEEE J. Solid-State Circuits.

[26] Beiert T., Schrickel J.W. (2019). Catheter ablation of cardiac arrhythmias. Herzschr Elektrophys.

[27] Venkata C., Ram S. (2019). Status of Renal Denervation Therapy for Hypertension. Circulation.

[28] Krum H., Schlaich M., Whitbourn R., Sobotka P.A., Sadowski J., Bartus K., Kapelak B., Walton A., Sievert H., Thambar S. (2009). Catheter-based renal sympathetic denervation for resistant hypertension: A multicentre safety and proof-of-principle cohort study. Lancet.

[29] Townsend R.R., Mahfoud F., Kandzari D.E., Kario K., Pocock S., Weber M.A., Ewen S., Tsioufis K., Tousoulis D., Sharp A.S.P. (2017). Catheter-based renal denervation in patients with uncontrolled hypertension in the absence of antihypertensive medications (SPYRAL HTN-OFF MED): A randomised, sham-controlled, proof-of-concept trial. Lancet.

[30] DiBona G.F., Esler M. (2010). Translational medicine: The antihypertensive effect of renal denervation. Am. J. Physiol. Integr. Comp. Physiol..

[31] Charthad J., Weber M.J., Chang T.C., Arbabian A. (2015). A mm-Sized Implantable Medical Device (IMD) With Ultrasonic Power Transfer and a Hybrid Bi-Directional Data Link. IEEE J. Solid-State Circuits.

[32] Mirbozorgi S.A., Yeon P., Ghovanloo M. (2017). Robust Wireless Power Transmission to mm-Sized Free-Floating Distributed Implants. IEEE Trans. Biomed. Circuits Syst..

[33] Lazzi G., DeMarco S.C., Liu W., Weiland J.D., Humayun M.S. (2003). Computed SAR and thermal elevation in a 0.25-mm 2-D model of the human eye and head in response to an implanted retinal stimulator-Part II: Results. IEEE Trans. Antennas Propag..

[34] Gosalia K., Lazzi G. (2004). SAR distribution and thermal elevation in a human head model due to the operation of the data telemetry link and implanted chip in a retinal prosthesis. IEEE Antennas Propag. Society Int. Symp..

[35] Elwassif M.M., Kong Q., Vazquez M., Bikson M. (2006). Bio-heat transfer model of deep brain stimulation-induced tempera-ture changes. J. Neural Eng..

[36] Quarteroni A., Formaggia L., Veneziani A. (2006). Cardiovascular mathematics. Proceedings of the International Congress of Mathematicians.

[37] Stroud J.S., Berger S.A., Saloner D. (2001). Numerical Analysis of Flow Through a Severely Stenotic Carotid Artery Bifurcation. J. Biomech. Eng..

[38] Chhabra R.P., Richardson J.F. (1999). Non-Newtonian Flow in the Process Industries: Fundamentals and Engineering Applica-Tions.

[39] Demi M. (2014). The Basics of Ultrasound. Compr. Biomed. Phys..

[40] Cheng A.H.-D., Cheng D.T. (2005). Heritage and early history of the boundary element method. Eng. Anal. Bound. Elem..

[41] COMSOL Multiphysics (2018). Introduction to COMSOL multiphysics®.

[42] Hoskins P.R. (2008). Simulation and Validation of Arterial Ultrasound Imaging and Blood Flow. Ultrasound Med. Biol..

[43] Shaik E. (2007). Numerical Simulations of Blood Flow in Arteries Using Fluid-Structure Interactions. Ph.D. Thesis.

[44] Jahanyfard E., Firoozabadi B., Chegini A.G. (2007). Computational Simulation of Non-Newtonian Blood Flow in Carotid Bifurcation for Investigating the Various Rheological Blood Models.

[45] Valencia A., Zarate A., Galvez M., Badilla L. (2006). Non-Newtonian blood flow dynamics in a right internal carotid artery with a saccular aneurysm. Int. J. Numer. Methods Fluids.

[46] Roland A. (1985). Model Research: The National Advisory Committee for Aeronautics, 1915–1958.

[47] Jiji L.M. (2009). Heat Convection.

[48] Kelly G. (2006). Body temperature variability (Part 1): A review of the history of body temperature and its variability due to site selection, biological rhythms, fitness, and aging. Altern. Med. Rev. A J. Clin. Ther..

[49] Kelly G.S. (2007). Body temperature variability (Part 2): Masking influences of body temperature variability and a review of body temperature variability in disease. Altern. Med. Rev. A J. Clin. Ther..

[50] Kollmann C. (2015). Diagnostic Ultrasound Imaging: Inside Out (Second Edition). Ultrasound Med. Biol..

